# The Establishment and Development of Neurosurgery Services in Papua New Guinea

**DOI:** 10.1007/s00268-015-3268-1

**Published:** 2015-10-19

**Authors:** W. Matui Kaptigau, Jeffrey V. Rosenfeld, Ikau Kevau, David A. Watters

**Affiliations:** Department of Surgery, Port Moresby General Hospital, Port Moresby, Papua New Guinea; Department of Surgery, University of Papua New Guinea, Port Moresby, Papua New Guinea; Department of Neurosurgery, Alfred Hospital, Melbourne, VIC Australia; Department of Surgery. Central Clinical School, Monash University, 6th Floor, The Alfred Centre, 99 Commercial Road, Melbourne, VIC 3004 Australia; F. Edward Hébert School of Medicine, Uniformed Services University of the Health Sciences, Bethesda, MD USA; School of Medicine and Health Sciences, Deakin University, Geelong, Australia

## Abstract

**Background:**

Papua New Guinea (PNG) is a developing Pacific Nation of 7.3 million people. Although neurosurgery training was introduced to PNG in the year 2000, it was in 2003 that a neurosurgery service was established. Prior to this time, neurosurgery in PNG was performed by general surgeons, with some assistance from visiting Australian neurosurgeons. Neurosurgical training was introduced to PNG in 2000. The model involved a further 3 years of training for a surgeon who had already completed 4 years of general surgical training. We aim to review the output, outcomes and impact achieved by training the first national neurosurgeon.

**Methods:**

The data on activity (output) and outcomes were collected prospectively from 2003–2012. Ongoing mentoring and continuing professional development were provided through annual neurosurgical visits from Australia. There were serious limitations in the provision of equipment, with a lack of computerized tomographic or MR imaging, and adjuvant oncological services.

**Results:**

There were 1618 neurosurgery admissions, 1020 neurosurgical procedures with a 5.74 % overall mortality. Seventy percent of cases presented as emergencies. There were improved outcomes, particularly for head injuries, whilst hydrocephalus was managed with an acceptable morbidity and revision rate.

**Conclusions:**

The training of a neurosurgeon resulted in PNG patients receiving a better range of surgical services, with a lower mortality. The outcomes able to be delivered were limited by late presentations of patients and lack of resources including imaging. These themes are familiar to all low- and middle-income countries (LMICs) and this may serve as a model for other LMIC neurosurgical services to adopt as they consider whether to establish and develop neurosurgical and other sub-specialist surgical services.

## Introduction

Papua New Guinea (PNG) is an independent country with 7.3 million people and a population growth rate of 2.1 % [1]. It is a low-middle income country (LMIC) with a GDP of US$15.41 billion [2], a per capita total health expenditure of US$97 and life expectancy of 65 years in females and 60 in males [3]. PNG’s health statistics are poor, when compared with other Pacific Island nations, with PNG reporting infant mortality rates at 47 deaths per 1000 live births and maternal mortality at 220 per 100,000 live births [3].

The capacity of national surgeons to provide surgical care has evolved rapidly over the three decades since the national Masters of Medicine (MMed) program through the University of Papua New Guinea (UPNG) produced its first graduates [4–6]. Once a critical number of general surgeons had graduated, further training in surgical sub-specialties was offered from 1994, leading to the award of a sub-specialist surgical diploma [4]. The first author (the late WMK) was the inaugural graduate from this program in neurosurgery.

Neurosurgical services are essential if PNG is to meet the health needs of its people as aspired to in the National Health plan 2010–2020 [7]. In an international survey conducted in 2004, the World Health Organization (WHO) found the median number of neurosurgeons per 100,000 population to vary from 0.03 to 0.97 in low- and high-income countries, respectively [8]. There has only been one neurosurgeon in PNG over the last decade since the establishment of the first neurosurgery unit in Port Moresby General Hospital (PMGH) in 2003, though visiting neurosurgeons from Australia and the People’s Republic of China have supplemented the service during specialist visits or specialist placements, respectively, since 2005. General surgeons in the provincial hospitals provide neurosurgical care for victims of trauma, infection and some congenital disorders such as myelomeningocele. PMGH is the National Referral Hospital for PNG, a country with 21 provinces each with its own Provincial Hospital, most of which are staffed by general surgeons. PMGH is a major teaching hospital of the University of PNG Medical School. Complex neurosurgery cases are sent to PMGH from other provinces. Australian neurosurgeons visit briefly twice a year to extend the range of surgery provided and build capacity by transferring knowledge and skills to local surgeons and nurses [9–14]. The PNG neurosurgery service has evolved without a major technology acquisition. A computerized tomographic (CT) scanner was available in a private hospital throughout the period but only became available at PMGH in 2008 through a donation, and required a co-payment for cost-recovery. No in-country magnetic resonance imaging (MRI) or radiotherapy services were available.

We aimed to review the workload, outcomes and impact achieved by training a neurosurgeon and setting up a neurosurgical unit at PMGH. The neurosurgical unit delivered emergency and elective neurosurgical care to a low-income population in a resource poor setting. The data reported here were based on a prospective audit over the first decade of the unit.

## Materials and methods

The neurosurgical service was established in 2003, following the first neurosurgeon’s qualification (WMK). The neurosurgeon was still required to lead a general surgical unit, participate in the on-call roster as well as providing a specialist service to neurosurgical patients. The hospital already had specialist surgical units in orthopaedics, ENT, maxillo-facial surgery and ophthalmology. Other general surgical units had sub-specialist interests in Urology, Head & Neck and Paediatric Surgery. The neurosurgery unit participated in regular audit and peer review meetings through the department of surgery. Autopsy results were correlated when performed, though in PNG there is often cultural and family reluctance to consent. Annual audit reports were provided to the other authors for professional development reviews. The data on activity (consultations, admissions and operations) and outcomes (in-patient mortality, infection rates, shunt complications etc.) were collected prospectively over a 10 year period from 2003 to 2012. Ongoing mentoring and continuing professional development were provided by visiting Australian neurosurgeons.

There were significant challenges resourcing the neurosurgical service. Many items of equipment often deemed essential for the conduct of neurosurgery in high-income countries were not available in PNG during this period. These included intracranial pressure monitoring devices, a complete set of neurosurgical operating room equipment including micro-instruments, neurosurgery operating table, modern bipolar coagulation, stereotactic frame, cavitron ultrasonic surgical aspirator (CUSA), neuroendoscopy and MRI.

The diagnosis of pituitary tumours was imprecise because hormone assays were not available. Transsphenoidal instruments were not available so all pituitary surgery was performed transcranially. Arginine vasopressin (DDAVP) for the treatment of diabetes insipidus was not available. The availability of chemotherapy drugs was limited and radiotherapy was not available in Port Moresby at any time, nor in the country until 2008.

Skull base tumours and sellar/parasellar lesions were referred overseas (usually to Australia) when deemed operable, but only if the family could bear the cost or a charitable organisation could fund treatment. Severe traumatic brain injury (TBI) cases were often not admitted to the intensive care unit because of a lack of beds or ventilators. Many patients with myelopathy lacked a definitive diagnosis due to inconclusive or unavailable CT myelograms.

Pre-hospital issues such as poor communication networks, lack of sealed roads and limited rural ambulance services in remote areas, meant TBI and other urgent neurosurgery patients were delayed in their arrival to PMGH. Many patients with neurosurgical problems were frequently treated by village shamans, and so presented late to PMGH. Those patients assessed by general surgeons and physicians in provincial hospitals, and who had advanced pathology, were usually advised to have conservative management and return to their village.

The hospital had many infrastructural issues and shortages. Hospital-initiated postponements of surgery occurred in 27.3 % of planned procedures. The three leading causes were equipment failure or deficiency, the patient being unfit for surgery and a shortage of operating room nursing staff. Perioperative mortality was defined as deaths that occurred within 30 days from the date of surgery.

## Results

Over the decade, the neurosurgical service provided 3626 consultations, managed 1618 admissions and performed 1020 operations (see Table 1). The most frequent pathology was TBI (36 %), followed by hydrocephalus (21 %), brain mass lesions (10 %) and scalp lumps (6 %). Spinal lesions (6.7 %) were dominated by patients with infection and myelopathy who presented with spinal cord compression and were treated on the basis of CT myelography. One-fifth of the spinal referrals were related to the congenital deformities of the spine. Disorders of the peripheral nerves accounted for only 1.3 % of referrals. The majority of admissions were emergencies (70 %). Despite the neurosurgical capability in the unit, only 31 % of 5347 admissions and 28 % of 3754 procedures performed were neurosurgical. The outcomes are shown in the Table 2.

**Table 1:** Diagnoses, consultations, admissions and procedures in the neurosurgical unit, Port Moresby General Hospital, 2003–2012

Pathology/condition	Consultations	Admissions	Procedures
Cranium			
Traumatic brain injury	1309	1067	442
Scalp suture			19
Cranioplasty for defect			7
Hydrocephalus	758	159	134
Ventricular tap			88
Mass lesions	361	174	81
Dermoid and scalp lumps	227	27	63
Headache	149		
Neurological manifestations of other diseases	119		
Dysraphism/congenital	111	23	38
Vascular and other lesions	102	33	3
Infections including TB	66	57	62
Change of dressing			15
Epilepsy	54		
Cerebrovascular accident	22		
Craniosynostosis	13		
Spine			
Infection including TB	70	36	20
Myelopathy	66		
Trauma	54	4	3
Congenital	50	9	9
Back pain and degenerative	38		
Mass lesion	4	9	9
Other spinal procedures		10	10
Peripheral nerve	53	10	17
Total	3626	1618	1020

Not all cases admitted had a prior consultation, and not all patients with a procedure were admitted as some were operated as day patients

**Table 2:** Outcomes of neurosurgical admissions (1618) and procedures (1020) performed in the Port Moresby Neurosurgical Unit, 2003–2012

Parameters measured	2003–2012	Outcome
N/S operations death rates	1020	56 (5.49 %)
Death rates for N/S admissions	1618	243 (15 %)
Death rates for cranial tumour operation	Operations 81	Mortality 7 (8.6 %)
Unplanned re-operation rates for cranial tumours	Operations 81	Re-operations 4 (4.9 %)
VP shunt operation—annual complication rate	Operations 134	Complications 22 (16.42 %)
VP shunt operation infection rate—cumulative	Operations 134	Infection 12 (8.9 %)
Overall mortality rate for VP shunt operation	Operations 134	Mortality 6 (4.5 %)
TBI mortality rates for mild, moderate and severe cases	Admissions (1067)	Mortality 171 (16 %)
Mild	541	18 (3.33 %)
Moderate	194	49 (35.26 %)
Severe	332	104 (31.33 %)
Nosocomial infection rates for all operations	Operations 1020	Infection 13 (1.27 %)

*N/S* neurosurgery, *TBI* traumatic brain injury, *VP* ventriculoperitoneal

Mortality for all neurosurgical patients was 15 % (Table 2). There were 56 (5.49 %) deaths following 1020 neurosurgical procedures. The Perioperative Mortality Rate (POMR) for all procedures performed by the neurosurgical unit, including the 2742 general surgical procedures was 2.3 %.

The clean wound infection rates (5 %) comprised predominantly VP shunt insertion (8.9 % of all shunts) or brain tumour operations. The nosocomial infection rate was 0.5 % for all admitted patients and 1.27 % for those that had neurosurgery and included hospital-acquired pneumonia inpatients on ventilators.

### Traumatic brain injury

There were 1067 patients admitted with TBI over 10 years (Table 2). Surgery was performed on 419 of these patients, of which 185 were for compound skull fractures. Closed TBI cases comprised 234 with localising signs requiring emergency surgery to evacuate a haematoma. Some cases of closed TBI had emergency burr holes without CT scans. In those cases, burr holes were first exploratory and then sometimes therapeutic, being converted to either craniectomy or craniotomy depending on the size and location of haematoma. After the introduction of a user-pay CT scanner in the public hospital, operations for closed TBI were more likely to be guided by its use (56 cases). There were 541 mild [Glasgow Coma Scale (GCS) 13–15], 194 moderate (GCS 9–12) and 332 severe (GCS ≤ 8) TBI cases. Of the 332 severe cases, only 113 were ventilated. Of the 167 TBI deaths, only 54 cases had CT scans. Patients who died from TBI without a CT scan were recommended for post-mortem to identify missed but correctable pathology. Post-mortems were performed on only 76 cases, as relatives often refused permission despite cases being referred to the coroner. An intracranial haematoma was found in 28 of the 76 autopsies, the remainder showing diffuse brain injury and/or oedema. Assaults were the leading cause of death on arrival (DOA), followed by motor vehicle accidents (MVA).

Of the post-operative deaths (86) there were 51(59.3 %) deaths following surgery for TBI. TBI also accounted for 51.6 % (176/341) of all deaths for those admitted to the neurosurgery unit. During the decade, the mortality of severe TBI fell from 48.8 % (2003) to 33.3 % (2012). Case fatality rates (CFR) of those who presented with moderate TBI cases were 35 % over the decade and 3.3 % for mild TBI (Table 2).

### Hydrocephalus

Of 231 patients with hydrocephalus, there were 213 children and 18 adults. We had 104 (45 %) that were due to presumed aqueduct stenosis based on imaging. This was mainly CT with ultrasound being used in infants. Seventy (38 %) were due to infection, 26 due to tumour and 6 to non-specified causes that were not easily diagnosed due to limitations of imaging studies. There were 134 VP shunt operations (35 revisions) performed on 111 patients with hydrocephalus. They were inserted only when there was no evidence of active infection. There were 12 infections complicating 134 ventriculo-peritoneal (VP) shunts over the decade (8.9 %).

### Brain mass

There were 236 brain mass lesions diagnosed by CT scan. The leading causes of brain mass lesions were 74 intracranial neoplasms, 28 abscesses and 13 tuberculomas; the latter all subsequently were confirmed at surgery. Only 5 of 74 neoplasms presented with blindness, compared to 10 out of 13 cases with TB. Three required VP shunts inserted for hydrocephalus prior to surgery for their neoplasm. The post-operative mortality for patients with brain tumours was 9.9 %. There were also 33 cases of dermoid cysts of the scalp excised. These cysts were often large and unsightly.

### Spine trauma

There were 202 spinal injuries. The commonest causes (44.6 %) were falls, usually from picking fruit from trees, followed by motor vehicle accidents (MVA) (36.6 %). Most MVA cases arose from travel in open-backed vehicles. Other causes of spinal trauma were assaults (11.4 %) and sports (5 %). The spinal region involved in these injuries included 119(61 %) cervical, 53(27.2 %) lumbar spine, 19(9.7 %) thoracic spine and 4(2 %) multi-level injuries, usually of the thoraco-lumbar spine. There were 4 deaths from cervical spine injuries. All mechanically unstable cervical spine injuries had cervical traction whereas thoraco-lumbar spine injuries received bed rest. Open reduction and internal fixation was performed by the orthopaedic surgeons in selected cases.

### CNS and spine infection

Spinal tuberculosis (TB) (66), brain TB (33) and brain abscess (28) were the leading admissions due to infection. Infection accounted for 33.9 % of brain mass lesions requiring surgery. Of these, 22.3 % were due to pyogenic brain abscess, 11.6 % brain TB and 2.7 % cryptococcoma. Surgery was performed on 13 of 33 cases with brain TB with one post-operative death. The remainder improved with TB treatment and did not require surgery.

There were 22 pyogenic brain abscesses drained with two deaths, a mortality rate of 9 %. Six improved on antibiotics. The remainder of the brain mass lesions were neoplastic (66 %).

Infection was the commonest cause of non-traumatic spinal cord compression with 20/36 (55.6 %) requiring decompressive surgery. The 16 remaining cases of spinal cord compression requiring surgery were eight with arachnoiditis from non-specific inflammation and eight neoplasms.

There were 20 cases of TB of the thoracic spine that had decompressive surgery: 11 with an anterior approach via thoracotomy and nine posterior approaches via costotransversectomy. These were operated on without complications and all recovered ambulation with bladder and bowel control upon discharge. The management of spinal TB was based on guidelines for PNG developed by the authors [15]. Surgery is indicated in cases with bladder or bowel incontinence or muscle power less than grade 3/5. Anterior decompression was performed in 11/20, utilising a thoracotomy, vertebrectomy and adding an autologous rib graft to buttress the defect. Forty-six patients with spinal TB (70 %) improved on chemotherapy and required no surgery.

### Other spine lesions

There were an additional 57 cases with spinal pathology and neurological impairment that required no surgery, including myelopathy of unknown aetiology (32), spinal degeneration (17) and neoplasms (8). The cause of myelopathy was sometimes not established because of lack of investigation. For example, only 12 cases of myelopathy were able to have a CT myelogram, whilst four went overseas for MRI. The cases of spinal degeneration were due to osteoarthritis (11), lumbar spondylolisthesis (3) and one each of stenosis, spondylosis and disc protrusion in the lumbar spine. Surgery was performed in one case of osteoarthritis with cervical cord compression. The eight cases of spinal neoplasm were due to lymphoma, with one each of neurofibroma, schwannoma, neuroblastoma and metastasis variably affecting the thoracic spine. All had decompressive laminectomy except two patients with lymphoma who had biopsy only. There was improvement in neurological function for those patients presenting with incomplete neurological deficit or in those with a shorter history prior to presentation.

### Congenital cranial and spinal pathology

Congenital cranial lesions were predominantly due to dermoid cysts (33) and encephaloceles (30). Most of the encephaloceles were fronto-nasal or ethmoidal, but there were three posterior and one at the vertex. Ten patients with encephalocele had surgery. The rest had no surgery due to lack of CT scan imaging (8), inadequate equipment (2), referral abroad (1) or refusal of treatment (9). There were 15 cases of spinal dysraphism with 6 cases operated on, comprising three cases of lipomyelomeningocele and one each of spinal lipoma, tethered cord and myelomeningocele.

## Discussion

For over a quarter of a century since PNG’s independence in 1975, the neurosurgical service offered by the country’s General Surgeons was rudimentary [5]. Pioneering surgeon Ken Clezy, the inaugural Professor of Surgery who had received some neurosurgical training in Australia, operated on meningiomas and pituitary tumours but only case reports were published [5]. The text *Neurosurgery in the Tropics* was based on the authors’ experience in PNG and described the management of common neurosurgical problems where patients are managed despite limited resources, often without access to CT scanning [14]. From 2003, neurosurgical activity reports were provided to PMGH and the PNG Department of Health but without receiving any official feedback. It was only following the establishment of a neurosurgery unit in 2003 under the first author (WMK), that early results were presented in a focus issue of the PNG Medical Journal in 2007. This did not describe the outcome and impact of the neurosurgical training as a whole [16].

The Lancet Commission on Global Surgery advocates for provision of safe, affordable and timely surgical and anaesthesia care for all [17]. Provision of specialist surgery is an integral component. Neurosurgery is not an expensive luxury, but enables emergency and essential operations to be done. The range of conditions treated, together with outcomes shows what can be achieved by training a specialist neurosurgeon. It also demonstrates how, despite limited equipment, the development of specialist skills in neurosurgery improves outcomes for the common conditions encountered, not only for TBI, but also for hydrocephalus, intracranial mass lesions, congenital anomalies and spinal cord and cauda equina compression. The training of a qualified general surgeon as a neurosurgeon demonstrates the success of the PNG model for sub-specialty training [4]. The combination of sub-specialist and general surgeon, in a country with a shortage of surgeons, has resulted in the unit continuing to manage general surgical emergencies and share in the on-call roster. Despite the risk of burnout this was the only model to which the National PNG Department of Health, faced with many demands for limited manpower resources, gave its support.

Clearly, the lack of equipment, infrastructural challenges and the need to also provide some cover in general surgery compromises the ability and time for a neurosurgeon to provide a comprehensive neurosurgical service. However, the falling mortality from TBI, provision of VP shunts with an acceptable infection and revision rate and the success of restoring lower limb, bladder and bowel function to patients with spinal decompression all attest to the impact of the service on the health of the community. It must be recognised that many more patients throughout PNG would have benefited had there been a health system that could efficiently transfer those cases unable to afford their own airfares. The POMR is a global indicator of access to and safety of surgery and anaesthesia and was acceptable in this audit [18].

The audit of cases provides important local epidemiological data on what is common and what conditions might present to the hospital. For example, the incidence of myelomeningocele cases is relatively low for a developing country. This may be because these infants were not referred or sent to the PMGH. Fronto-nasal encephaloceles are more common than the occipital location, the latter being more prevalent in parts of Asia [9]. Cerebrovascular disease is currently uncommon but is likely to increase as the adoption of Western diets increases. Degenerative spinal disease is still uncommon. The audit also shows the value of recording and reporting surgical outcomes, and how these can identify opportunities for improvement. Further improvement in TBI outcomes will be in part dependent on improving pre-hospital transport. The golden hour is usually missed in PNG, even in Port Moresby [19]. Greater access to better imaging will improve decision making, and greater capacity in intensive care will improve post-operative care.

The range of cases treated and the training received proves the value of establishing a network in the Pacific for neurosurgical support. JVR and his colleagues in Australia have provided mentorship and hospital attachments in Australia with the support of various scholarships [20]. The same model for neurosurgical training has also been recently adopted in Fiji [21].

During the second part of the 10-year period, 2003–2012, a second PNG neurosurgeon was also successfully trained using the same model. The two neurosurgeons also provided the neurosurgical education for doctors and general surgeons in training, ensuring that PNG’s postgraduate training in general surgery prepared graduates for the common neurosurgical presentations they would face in PNG’s 21 provinces. Sadly, the first author of this paper developed metastatic cancer and recently died. This paper not only highlights what can be achieved with neurosurgical training, but is also a tribute to what Dr. William Kaptigau (1963–2014) achieved in just one decade of neurosurgical practice.

Alternate models of neurosurgery training have evolved. Many LMIC have an established neurosurgery service with the senior neurosurgeons often having been trained in more advanced countries [22]. These neurosurgeons train the next generation of neurosurgeons ‘in-country’ although some of these trainees travel abroad for further training. Adeleye et al. describe a major Nigerian neurosurgery department with five senior registrars but ‘Nigerian neurosurgery still in its infancy five decades after its inauguration’ [23]. Adeleye et al. described a skull base surgery fellowship which included training time in Israel and further education in the United States [23].

Further models of training may include the development of short-term regional neurosurgery training centres [24], web-based e-Learning platforms [25], building capacity by training local health workers in basic and emergency neurosurgical procedures [26] and neurosurgical missions to teach particular skills [27]. Bernstein et al. surveyed neurosurgery trainees in Indonesia who felt their training in trauma was excellent but that it was lacking in the specialty areas [28]. Neurosurgeons in LMIC require a balanced training which focuses on the range of pathology they are likely to encounter in their own country and is set to the level of practice expected in that environment [28].
